# Interactions between pH, reactive species, and cells in plasma-activated water can remove algae†

**DOI:** 10.1039/d1ra07774k

**Published:** 2022-03-08

**Authors:** Ken Mizoi, Vicente Rodríguez-González, Mao Sasaki, Shoki Suzuki, Kaede Honda, Naoya Ishida, Norihiro Suzuki, Kazuyuki Kuchitsu, Takeshi Kondo, Makoto Yuasa, Akira Fujishima, Katsuya Teshima, Chiaki Terashima

**Affiliations:** Department of Pure and Applied Chemistry, Faculty of Science and Technology, Tokyo University of Science 2641 Yamazaki, Noda Chiba 278-8510 Japan terashima@rs.tus.ac.jp; Research Center for Space System Innovation, Research Institute for Science and Technology, Tokyo University of Science 2641 Yamazaki, Noda Chiba 278-8510 Japan; Instituto Potosino de Investigación Científica y Tecnológica (IPICyT), División de Materiales Avanzados Camino a La Presa San José 2055, Lomas 4a. Sección 78216 San Luis Potosí Mexico; Department of Applied Biological Science, Faculty of Science and Technology, Tokyo University of Science 2641 Yamazaki, Noda Chiba 278-8510 Japan; Research Initiative for Supra-Materials, Shinshu University Nagano 380-8553 Japan

## Abstract

Lightning strikes cause nitrogen to dissolve in water and form reactive nitrogen and oxygen species, which form natural fertilizers that can be absorbed through plant roots. Such processes during rainstorm events can be simulated by applying plasma to a solution. Plasma-activated water (PAW) has great potential as a source of various dissolved reactive chemical species. Different mixtures of species are produced using different solution compositions. Here, basil seeds were grown in PAW to prevent blooms of *Chlorella vulgaris* and ion chromatography and UV-vis spectroscopy were used to quantify reactive ions. NO_2_^−^, NO_3_^−^, and H_2_O_2_ were found to be key to the antialgal effect. Secondary reactive ions such as peroxynitrite (ONOO^−^, ONOOH) were also involved. The antialgal effect was strongly related to the pH around the algal cells. Acidification was predominantly caused by the generation of NO_2_^−^ and H_2_O_2_. After two weeks monitoring basil growth, the antifungal properties were preserved, few reactive oxygen species formed in the plasma zone, and only reactive nitrogen species were transformed into reactive peroxynitrite ions. The pH around the cells was determined using an iridium oxide microelectrode. The PAW antialgal mechanism depended on acidic conditions (pH 2.2, at which peroxynitrite can be generated) under which ONOOH penetrated the algal cell membranes, destroying the cells and preventing growth. This practical and sustainable PAW process allows a surprising amount of fertilizer to be generated with an antialgal effect that could be used in various eco-friendly agricultural processes under ambient conditions.

## Abstract 2

PAW is effective in inactivating microorganisms. We have measured a local pH to reveal the mechanism of algicidal effect in PAW. This is because protons pumped from the cell generate peroxynitrite around the cell to generate an acidic region.

## Graphical abstract

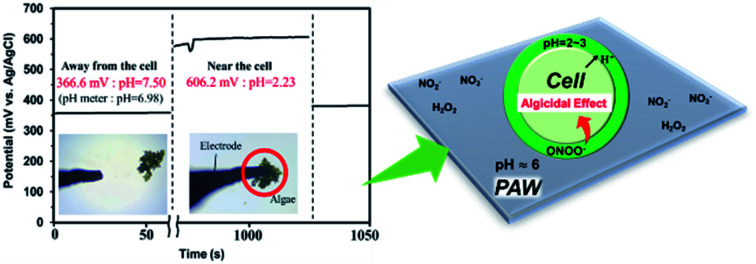

## Introduction

Low-temperature or cold atmospheric plasmas have been attracting attention in recent years in the fields of physics, chemistry, materials sciences,^1^ medicine,^2,3^ biology,^4^ and agriculture.^5–9^ Treating an aqueous solution with low-temperature plasma can give plasma-activated water (PAW), which contains active oxygen and nitrogen species^10^ and is an effective bactericide.^11–13^ PAW has been used in various applications,^14^ including sterilizing microorganisms,^15–17^ hardening fresh foods,^18,19^ promoting plant growth,^20,21^ and treating cancer.^22–25^ PAW has a strong, temporary algicidal effect on microorganisms, then returns to being fresh water after a certain time, so PAW is expected to be suitable for use in new environmentally friendly sterilization methods.^26^ PAW can be used to allow plants to be grown quickly regardless of the weather,^27^ so attention is being paid to using PAW to address food shortages caused by increases in the population, global warming, and other factors. However, algae readily grow in the solutions, cultivation tanks, and equipment used in plant-growing factories^28,29^ because of the moisture, light, and nutrients present. These problems, including cleaning labor and high running costs, partly explain why plant factories are not as popular as expected. Developing a liquid fertilizer in PAW that can be used to grow plants and prevent growth of algae and bacteria would allow clean and maintenance-free plant factories to be established and contribute to solving global food shortages, which will become more serious in future. More research into the use of PAW as an antibacterial liquid fertilizer is being performed.^30,31^ For example, Iwata *et al.* successfully produced PAW that simultaneously prevents microbes growing and supports plant growth.^6^ They concluded that organic compounds containing benzene rings give PAW with good antibacterial properties. This method gives PAW with antimicrobial properties and that supports plant growth, but the requirement for organic compounds containing benzene rings is not environmentally sustainable. Other ways of producing PAW that is algicidal but promotes plant growth have not been investigated. Many researchers have concluded that the algicidal effects of PAW are caused by peroxynitrite produced through reactions of active species in the PAW.^12,32^ Peroxynitrite, which is the conjugate acid of pernitrate, can oxidize, nitrate, and hydroxylate biomolecules under physiological conditions, which is why peroxynitrite is cytotoxic.^31^ Peroxynitrite has a high permeability coefficient of 8.0 × 10^−4^ cm s^−1^, which is comparable to the permeability coefficient of H_2_O_2_. This makes peroxynitrite an extremely effective oxidant for damaging bacteria.^26,33^ Peroxynitrite has strong antimicrobial properties at low concentrations.^34^ There are two forms of peroxynitrite, the peroxynitrite anion (OONO^−^) and peroxynitrous acid (ONOOH), which have an acid dissociation constant p*K*_a_ of 6.8 and will both be present in PAW, which is acidic or neutral.^15^ The formation of peroxynitrite in PAW has been investigated in many studies using fluorescent probes and UV-vis absorption spectroscopy to quantify, identify, and confirm the presence of peroxynitrite.^35^ PAW maintains its algicidal effect for a long time,^32,36–38^ up to one month. This was predicted because short-lived reactive oxygen species, which are important for peroxynitrite formation, are repeatedly re-generated in PAW. Peroxynitrite might be expected to be strongly cytotoxic to plants as well as bacteria because it dissociates readily under neutral conditions. However, PAW shows antibacterial effects but is not toxic to plants. This may be because reactive oxygen species and reactive nitrogen species will react under only certain conditions to produce peroxynitrite, which will directly attack only bacteria. PAW therefore has long and effective algicidal effects. The condition required for this effect is a highly acidic region in the vicinity of the cells to be attacked. Once the area around a cell becomes highly acidic, the appropriate chemical species in the PAW react to produce peroxynitrite, which penetrates and attacks the cell even if the bulk solution has a neutral pH. This makes PAW an effective bactericide. To prove this, it is necessary to determine the pH range at which peroxynitrite can be produced and to match this range to the pH around relevant cells. However, these pH ranges have not been determined, so it is not clear how peroxynitrite is produced and acts only on bacteria. We therefore aimed to prove that peroxynitrite in PAW is an important factor in the antialgal effect of PAW by directly measuring the pH near cells using microelectrodes in a neutral liquid, such as a hydroponic liquid fertilizer. In this study, (1) environmentally friendly PAW was prepared using nitrogen and water, (2) the antialgal effect of PAW was investigated, and (3) the mechanism involved in the antialgal effect of PAW against *Chlorella vulgaris* while allowing plants to grow was investigated focusing on the pH near the cells. The results confirmed that PAW produced using water and nitrogen is an ideal disinfectant that acts only on bacteria and microorganisms. The results provide a starting point for identifying the mechanism involved in the biocidal effect of PAW.

## Results and discussion

### Confirmation of the batch plasma emission state

**Fig. 1:**
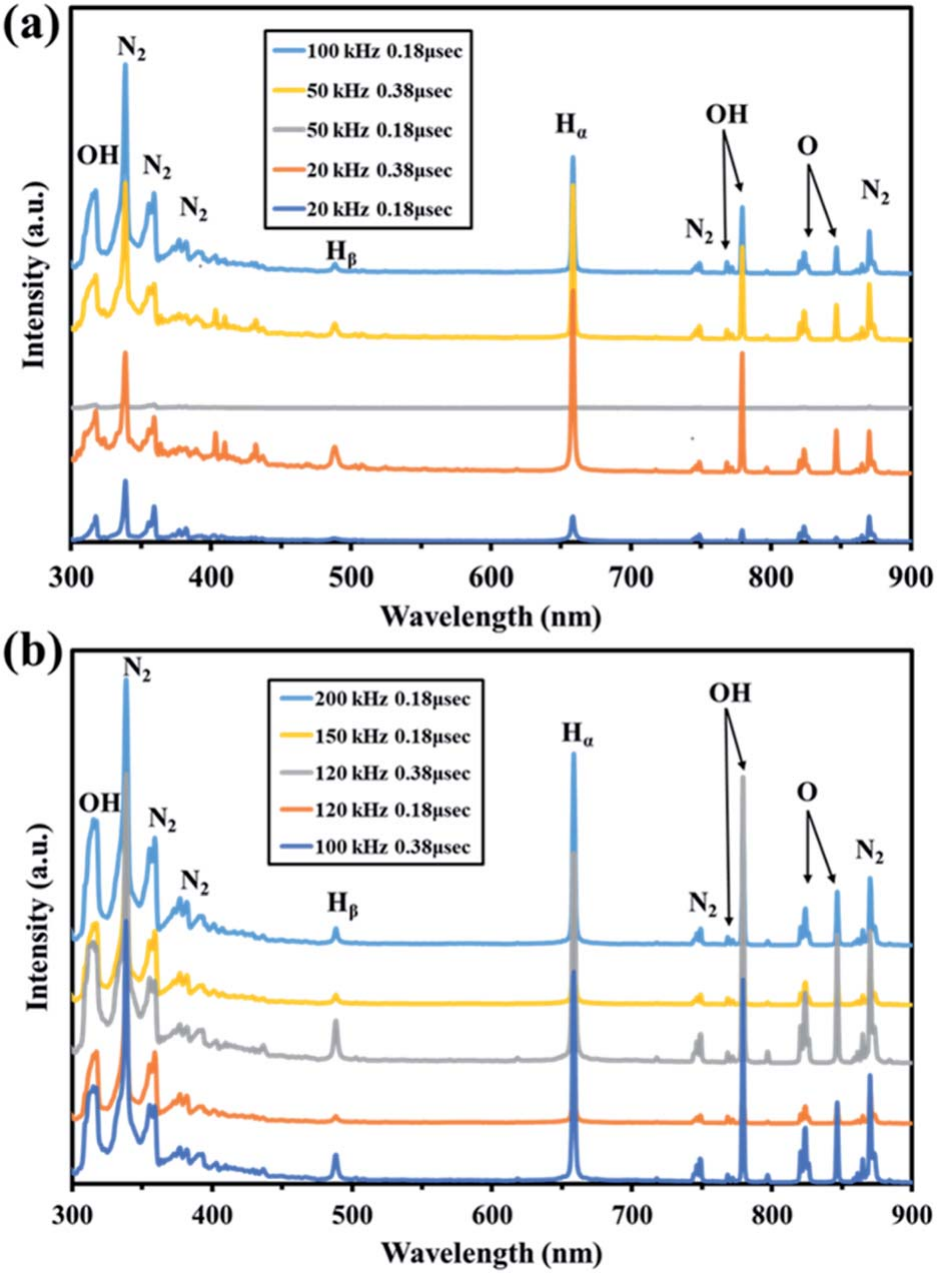
Visible spectra of plasma generated in the batch-type in-liquid plasma system. (a) No. 1–5. (b) No. 6–10.

A plasma emission analyzer was used in the range 300–900 nm to investigate the excited active species generated in nitrogen using a batch-type in-liquid plasma system using 10 conditions with different frequencies and pulse widths. As shown in Fig. 1, the main peaks were for water (*i.e.*, OH (317, 769, and 779 nm), Hα (656 nm), Hβ (486 nm), and O (823 and 846 nm)) and N_2_ (338, 357, 369–392, 749, and 870 nm).^39–41^ We assumed that the plasma decomposed water to give OH, O, and H. N_2_ peaks were also found. This suggested that reactive nitrogen species and reactive oxygen species formed in the solution. These results suggested that various reactive oxygen and nitrogen species were formed in the gas phase by the batch-type in-liquid plasma system and became fixed in the solution by coming into contact with the liquid. Unlike in the emission spectrum obtained by Mizukoshi *et al.*, only the short-wavelength region of the N_2_ spectrum was acquired using the batch method.^42^ This was because Mizukoshi *et al.* used air to generate plasma in water but we used N_2_, which gave plasma containing a large amount of nitrogen. The PAW was used not only as a cell and microbial disinfectant but also as a liquid fertilizer, so it was very important to fix a large amount of nitrogen in the solution to act as a nutrient source for the plants. The electron temperature was calculated from the Hα and Hβ peak intensities (Tables S1 and S2†). The electron temperature varied depending on the conditions but was ∼1 eV. An electron temperature of 15 000 K (1.29 eV) was previously found using the same measurement method for a batch-type in-liquid plasma system, so the value obtained in this experiment was considered to be reasonable.^43^ The electrodes melted or were lost in a short time when the frequency was increased above 120 kHz. We therefore used 100 kHz and 0.18 μs (line 5 in Table S1†), which gave the highest electron temperature and allowed a stable plasma treatment to be maintained for a long time.

### Investigation of PAW characteristics

**Fig. 2:**
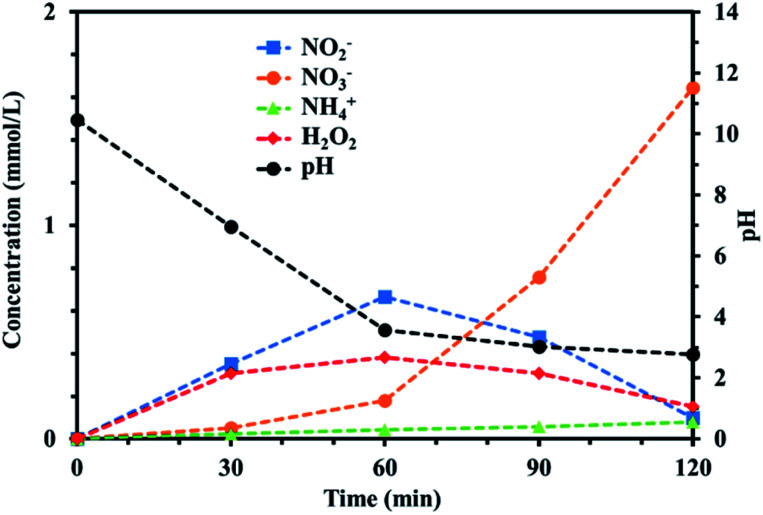
Temporal changes in the NH_4_^+^, NO_2_^−^, NO_3_^−^, and H_2_O_2_ concentrations during the plasma treatment. The pH change for reaction time is also plotted.

Ion chromatography measurements were made and UV-vis spectra acquired to quantitatively and qualitatively analyze the active species in the PAW produced using the batch-type submerged plasma method using nitrogen. The results are shown in Fig. 2. NO_2_^−^, NO_3_^−^, NH_4_^+^, and H_2_O_2_ were found to be produced in the PAW. The NO_2_^−^ and H_2_O_2_ concentrations continued to increase until 60 min after the treatment started, then the concentrations decreased. The NO_3_^−^ and NH_4_^+^ concentrations continued to increase as the treatment continued. NO_3_^−^ and NO_2_^−^ were probably produced through the dissociation of nitrogen oxide species generated in the gas phase of the plasma. The production of NH_4_^+^ suggested that oxidation and reduction reactions occurred simultaneously. The continuous increase in the NH_4_^+^ concentration indicated that NH_4_^+^ did not decompose but remained in the system. The reactions involved in the production of NH_4_^+^ are shown in eqn (1) and (2). Dissociation of nitrogen oxide species in the liquid phase would have proceeded as the treatment continued, and the pH decreased markedly as the NO_2_^−^, NO_3_^−^, and H^+^ concentrations increased. The reactions involved in these processes are shown in eqn (3)–(5).

1 N + 3H → NH_3_

2 NH_3_ + H^+^ → NH_4_^+^

3 NO_2_ + NO_2_ + H_2_O → NO_2_^−^ + NO_3_^−^ + 2H^+^

4 NO + NO_2_ + H_2_O → 2NO_2_^−^ + 2H^+^

5 3NO_2_^−^ + 3H^+^ → NO + NO_3_^−^ + H_3_O^+^

In particular, the NO_3_^−^ concentration increased for 60 min after the plasma treatment started but the NO_2_^−^ and H_2_O_2_ concentrations decreased markedly. We concluded that large amounts of H_2_O_2_ and NO_2_^−^ were produced during the first 60 min of treatment and that NO_2_^−^ was oxidized by H_2_O_2_ to give large amounts of NO_3_^−^ as the treatment continued. H_2_O_2_ is a strong oxidant under acidic conditions, so it was expected that H_2_O_2_ acted as an oxidant in the PAW system. In other words, H_2_O_2_ started to act as an oxidant when the pH decreased as the treatment time increased. The oxidizing ability of H_2_O_2_ increased markedly after 60 min, probably because the system had become acidic.

### Alga prevention experiment

**Fig. 3:**
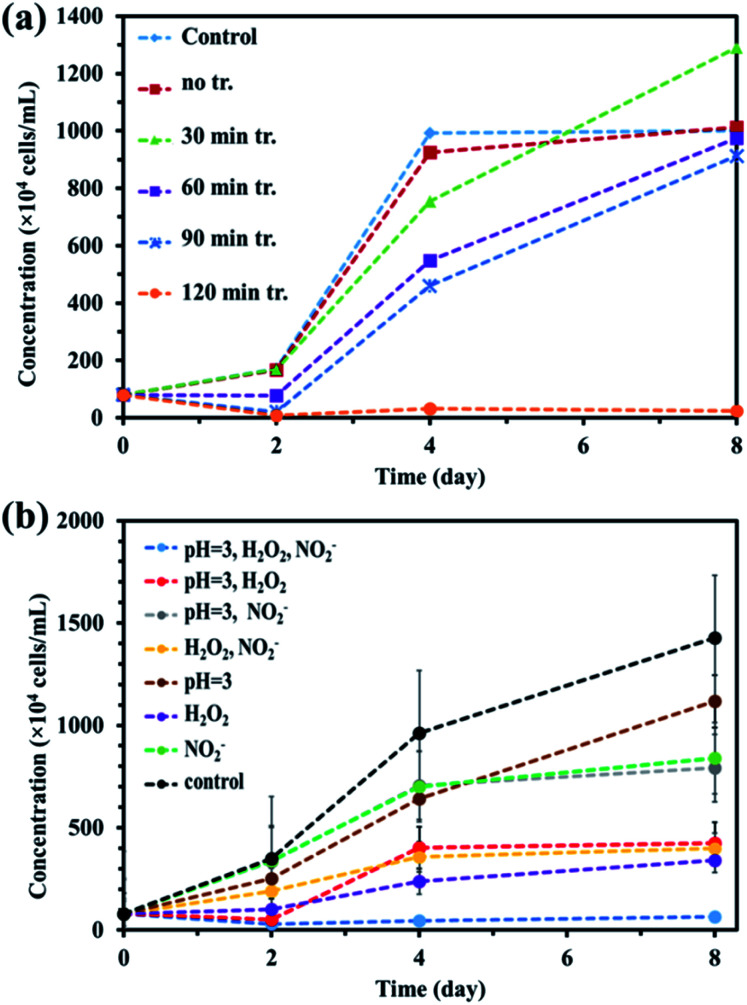
(a) Prevention of algae growth in plasma-activated water with the different plasma-treatment times and (b) prevention of algae growth in the mimic solutions with slightly different compositions based on plasma-activated water with 120 min treatment.

Alga prevention experiments were performed using untreated water and PAW produced for 30, 60, 90, and 120 min using a batch-type in-liquid plasma system and control samples. The results are shown in Fig. 3(a). The cell concentrations in the samples treated for 60, 90, and 120 min on the second day were lower than the initial concentration of 80 × 10^4^ cells per mL, indicating that treatment for >50 min effectively prevented algae growing. After 8 d, only the samples treated for 120 min contained a cell concentration lower than the initial concentration. These results indicated that a long-term algicidal effect only occurred in the sample treated for 120 min. The results shown in Fig. 3 indicated that the pH, NO_2_^−^, NO_3_^−^, and H_2_O_2_ were responsible for preventing algae growing in the sample treated for 120 min. PAW containing nitrogen oxides has previously been found to have an algicidal effect,^44^ and this has been found to be associated with synergistic effects between NO_2_^−^, NO_3_^−^, and H_2_O_2_. We investigated the factors contributing to preventing algae growing by focusing on the pH and NO_2_^−^, NO_3_^−^, and H_2_O_2_ concentrations in the PAW treated for 120 min that prevented algae growing for about one week. We prepared mimic solutions and used them to perform experiments to investigate the prevention of algal growth. The pH of each solution was adjusted by adding nitric acid. The NO_2_^−^ and H_2_O_2_ concentrations were 0.1 and 0.4 mmol L^−1^, respectively, which were the same concentrations as the PAW for 120 min. Seven solutions were prepared under the different conditions shown in Table 1, and algal growth in their solutions and the control were compared. The results are shown in Fig. 3(b). The algal cell concentrations were lower in the samples at pH 3 and containing NO_2_^−^ and H_2_O_2_ than the initial concentration of 80 × 10^4^ cells per mL after 8 d. This confirmed that algae were prevented from growing only under acidic conditions and in the presence of NO_2_^−^, NO_3_^−^, and H_2_O_2_. This suggested that algae were prevented from growing in the PAW by these four factors. Algae being prevented from growing under acidic conditions and in the presence of NO_2_^−^, NO_3_^−^, and H_2_O_2_ suggested that NO_2_^−^ and H_2_O_2_ react in an acidic solution to give peroxynitrite (ONOOH, ONOO^−^), which has a strong algicidal effect. ONOOH is formed through a reaction between O_2_^−^ and H_2_O_2_ under strongly acidic conditions, and dissociates into 
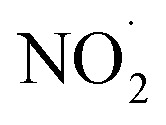
 and OH˙, which exert strong algicidal effects under neutral conditions.^45^ The reactions involved are shown in eqn (6)–(8).

**Table 1:** Preparing conditions of mimic solution according to plasma assisted water with 120 min treatment

Entry	Content		
	pH = 3	NO_2_^−^	H_2_O_2_
1	○	○	○
2	○	✗	○
3	○	○	✗
4	✗	○	○
5	○	✗	✗
6	✗	✗	○
7	✗	○	✗

Acidic conditions

6 NO_2_^−^ + 2H^+^ ↔ H_2_NO_2_ + ↔ NO^+^ + H_2_O

7 NO^+^ + H_2_O_2_ ↔ ONOOH + H^+^

Neutral conditions8
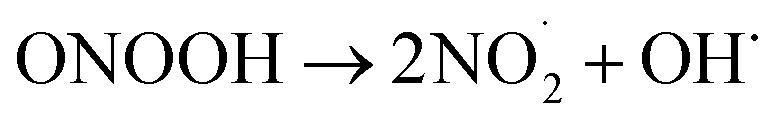


The interiors of cells are kept at neutral pH values, so we assumed that the interiors of the algal cells were at neutral pH values. The ONOOH generated in the PAW system could penetrate the membranes of an algal cell and dissociate into active algicidal species inside the cell, which would destroy the algal cell from within. ONOOH is produced through a reaction between NO_2_^−^ and H_2_O_2_, which both have longer lifetimes than many other active species. We therefore concluded that PAW has a long-lasting algicidal effect because the reaction between NO_2_^−^ and H_2_O_2_ occurs gradually.^6,32,38^ The aim of this study was to use PAW as a liquid fertilizer for growing plants while preventing the growth of algae. The possibility of using PAW for growing plants was investigated by performing basil growth tests. The results are shown in Fig. S1.† The results indicated that PAW can be used to grow plants while suppressing the growth of algae. We concluded that PAW is a liquid fertilizer that can be used to grow plants while preventing algae growing. It is very interesting that the algae were killed but the basil grew normally. We hypothesized that this may have been because, unlike for the algae, the pH near the basil cells was not acidic and peroxynitrite was not produced. One possible reason for this is that, like algal cells, basil cells will release protons through a proton pump, but the protons released will diffuse more quickly in all directions from basil plants than algae because basil plants are larger than algae. This means that peroxynitrite will not be produced near the basil cells. Another possible reason is that protons are consumed by basil plant cell walls during growth, preventing acidification around the cell and therefore preventing peroxynitrite forming. Plants grow by cell division and cell elongation. Each plant cell has a cell wall, which is acidified and softened by proton pumps in the cell membrane and then stretched (the acid growth theory).^46^ The proton pump in the cell membrane acidifies the cell wall, softens it, and stretches it. These possible reasons led us to predict that the pH near the cells of plants such as basil will decrease enough for ONOOH to be produced but that ONOOH is produced only near the algal cells. If the pH in the vicinity of the algal cells was found to become low enough for ONOOH to form it may be possible to firmly conclude that ONOOH in PAW destroys algal cells and plays an important role in the algicidal effect of the PAW. Microelectrodes were fabricated to allow the pH in the vicinity of algal cells to be measured to investigate this.

### Measurements of the pH in the vicinity of algal cells

**Fig. 4:**
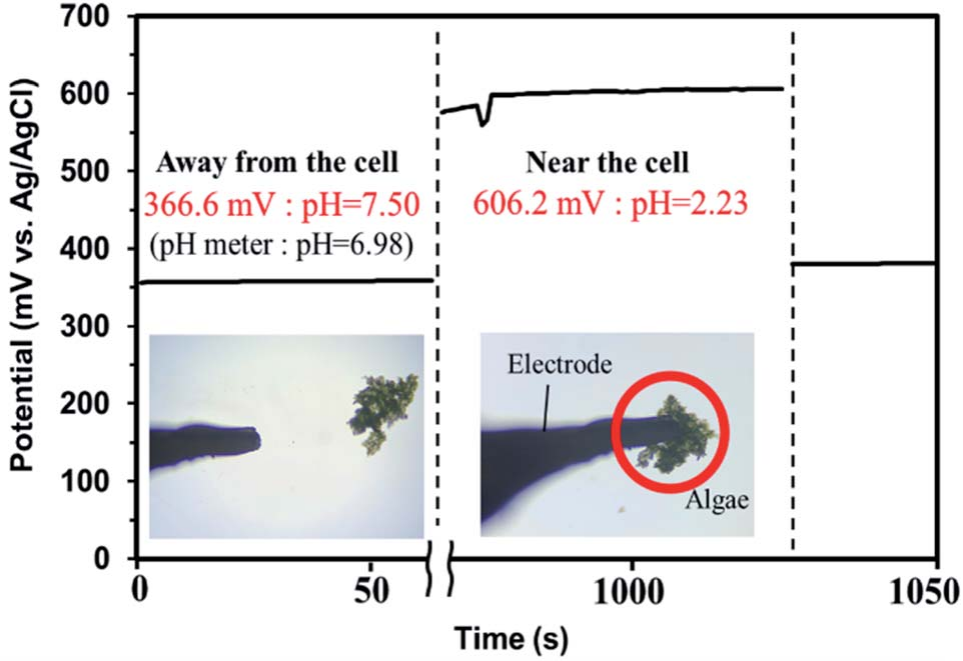
Cell proximity measurements performed using microelectrodes with iridium oxide electrodeposited on the tips.

**Fig. 5:**
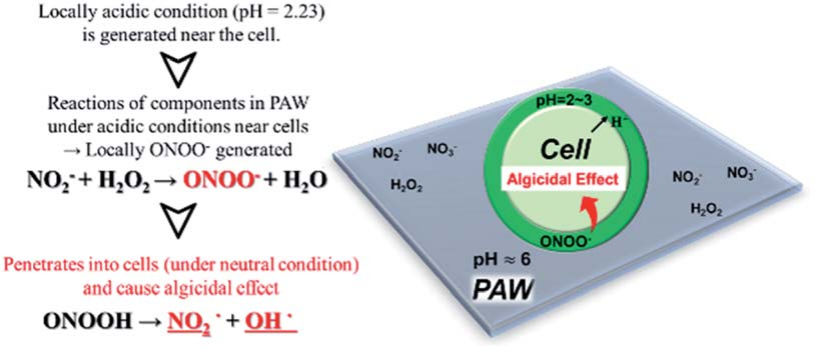
Scheme showing how plasma-activated water (PAW) is cytotoxic only to microorganisms.

The results of the algicide experiments and the experiments performed to investigate the factors involved led to the conclusion that the algicidal effect of PAW was caused by ONOO^−^ forming through a reaction between NO_2_^−^ and H_2_O_2_ in the PAW under strongly acidic conditions. ONOO^−^ dissociates into OH˙ and 
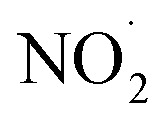
, which can penetrate the algal cells at neutral pH and kill the cells. We thought that the mechanism preventing algae growing could be determined by making direct observations of the pH near the algal cells. However, direct observations of the pH near cells in a liquid at neutral pH (*e.g.*, hydroponic nutrient solution) have not previously been made. First, microelectrodes for measuring the pH were fabricated and characterized. The characterization results are shown in Fig. S2.† A sigmoidal current–potential curve was found. This is a characteristic of microelectrodes and confirmed that the microelectrodes had been fabricated successfully. Next, stable pH-responsiveness even under strong acidic conditions was achieved by electrodepositing iridium oxide (an inorganic metal oxide) on the tip of each microelectrode. The current was measured using the fabricated microelectrode to perform cyclic voltammetry (CV), and the pH responsivity was evaluated. The results are shown in Fig. S3.† The slope of the straight line (*y* = −45.454*x* + 707.77) indicated that the pH response gradient was −45.45 mV pH^−1^ unit. The error was very low, confirming that the successfully fabricated electrode gave very reproducible results. The reversibility of the microelectrode measurement was assessed by measuring the open-circuit potential difference (OCP) when the microelectrode was moved from a neutral to an acidic solution and then back to a neutral solution. The results are shown in Fig. S4.† The OCP was almost constant at all pH values except approximately pH 3, suggesting that the microelectrode was reversible. The pH values found near algal cells using microelectrodes electrodeposited with iridium oxide are shown in Fig. 4. The OCP near the cells was 606.2 mV, which indicated that the solution was at pH 2.23 (calculated from the calibration curve). The UV-vis measurement results shown in Fig. S5† allowed the pH region in which ONOOH could be generated to be identified. The ONOOH concentration was calculated from the fluorescence intensity using a calibration curve (Fig. S6†). The results indicated that ONOOH formed slowly at approximately pH 2–3 and rapidly at pH 1. We concluded that the solution near an algal cell needed to between pH 1 and 3 for ONOOH to form and that the best performance was achieved at pH 1–2. The results suggested that the solution near each algal cell was at ∼pH 2 and that peroxynitrite was likely to be produced in the vicinity of each algal cell. The pH may have been low in the vicinity of each algal cell because algal cells have proton pumps in their cell membranes and use the electrochemical potential difference of protons to convert energy to allow metabolic activities. When a cell releases a proton, the pH in the vicinity of the cell will decrease because movement of a proton released from the cell surface will be rate-limited because of the small size of the cell (a 2–10 μm sphere). The results suggested that the acidic conditions in the vicinities of the cells caused a reaction between NO_2_^−^ and H_2_O_2_ (components of the PAW) to occur to produce peroxynitrite only in the vicinities of the cells. The peroxynitrite generated in the vicinities of the algal cells will have dissociated into 
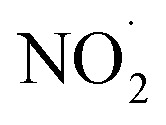
 and OH˙ (eqn (8)), which would have been transported to within the cells and killed the cells, as shown in Fig. 5. We concluded that the PAW system had a long-term and strong antialgal effect.

## Experimental

### Production of PAW using a batch-type in-liquid plasma system

In-liquid plasma was generated using a pulsed plasma system (MPP-HV04, supplied by Kurita Industries) in a batch-type in-liquid plasma device shown in Fig. S7.† Two electrodes were placed facing each other in the electrolyte solution, and an electric current was applied to the electrodes. This superheated the electrodes, and the liquid around them started to boil.^47,48^ The liquid produced bubbles in the water and plasma was generated within the bubbles. A hollow tungsten rod (outer diameter 3 mm, inner diameter 1 mm) was used for each electrode to allow N_2_ to flow through. Plasma was generated in the bubbles around the electrodes and in bubbles produced by N_2_ from outside the electrodes. The system was expected to prevent the temperature of the solution increasing and minimize wear-and-tear of the electrode. Each electrode was covered with an insulated tube (outer diameter 5 mm) placed so that the distance between the electrode surface and the inner surface of the insulated tube was 1 mm. N_2_ was passed through the tip of each hollow electrode at a flow rate of 100 mL min^−1^ so that the N_2_ directly encountered the plasma. The plasma treatment was applied to a K_3_PO_4_ solution (100 mg L^−1^, 350 mL; Sigma-Aldrich, St. Louis, MO, USA). K_3_PO_4_ is used as a liquid fertilizer when growing plants hydroponically. The solution was mixed using a pump to give a circulation rate of 250 revolutions min^−1^ to ensure uniform plasma treatment of the solution. The solution was kept at 3 °C using a Liebig cooler. A pulsed power supply was connected to the electrodes, and the plasma treatment was performed using 10 different conditions with different pulse frequencies and widths to allow the optimum power supply conditions to be identified. A plasma emission spectrometer (c7460; Hamamatsu Photonics) was attached to the batch-type plasma system and used over the wavelength range 300–900 nm to investigate excited active species in the plasma. The Hα and Hβ peak intensities and the electron temperature measured using the emission analyzer were calculated. The results led to PAW being produced using a pulse frequency of 100 kHz, a pulse width of 0.18 μs, and a voltage of 4 kV, which allowed stable plasma treatment to be performed for a long time. PAW was produced by treating water for 30, 60, 90, and 120 min at a pulse frequency of 100 kHz, pulse width of 0.18 μs, and voltage of 4 kV. Once the PAW was prepared, the pH of the liquid was measured using a pH meter (D-210PD; HORIBA). Ion chromatography measurements and UV-vis spectra were acquired to allow qualitative and quantitative analyses of the active species in the PAW to be performed. The ion chromatograph was used with a suppressor current of 30 mA for anions and of 60 mA for cations, a flow rate of 1.5 mL min^−1^ for anions and of 1.0 mL min^−1^ for cations, and a column temperature of 30 °C for both anions and cations. The ion chromatograph was a Dionex ICS-1100 system (Thermo Fisher Scientific, Waltham, MA, USA) fitted with a Dionex AS12A column for anions or a Dionex CS12A column for cations, and the suppressor was a Dionex ERS-500 system. The H_2_O_2_ concentration in the PAW was determined using a colorimetric method.^49,50^ For liquid A, 1 g of sodium hydroxide (Wako Pure Chemical Industries, Osaka, Japan), 33 g of potassium iodide (Wako Pure Chemical Industries), and 0.1 g of heptamolybdic acid hexammonium tetrahydrate (Wako Pure Chemical Industries) were added to a 500 mL flask, then the flask was filled with water to dissolve the salts. Liquid B was prepared by adding 10 g of potassium hydrogen phthalate (Tokyo Chemical Industry, Tokyo, Japan) to a 500 mL flask, then the flask was filled with water to dissolve the salt. Solutions A and B were mixed with a sample at a ratio of 1 : 1 : 1, and the absorption intensity at a wavelength of 350 nm was determined using a UV-vis spectrophotometer. The scanning speed was 100 nm min^−1^ and the scanning range was 500–300 nm. The UV-vis spectrophotometer was a V-670 system (Japan Spectroscopic Co).

### Alga prevention experiment

Experiments in which algae were prevented from growing were performed using *Chlorella vulgaris* (NIES-2170) purchased from the National Institute for Environmental Studies. A 5 mL aliquot of 1/10 Hoagland solution and 0.5 mL of a *Chlorella vulgaris* suspension were added to an autoclaved test tube to give a *Chlorella vulgaris* concentration of 80 × 10^4^ cells per mL. The Hoagland solution was used to reproduce a growing solution used in a plant growing factory. The Hoagland solution was prepared using stock solutions of six components, as shown in Table S3.† The stock solutions were added to 1 L of ultrapure water in the appropriate ratios to give a 1/10 Hoagland solution at pH 5.6–5.8. The solution was autoclaved at 120 °C before being used in the experiments. Six samples were used in the experiments. These were the unmodified culture medium (control), untreated K_3_PO_4_ solution, and PAW treated for 30, 60, 90, and 120 min. The culture medium was liquid C medium, purchased from the National Institute for Environmental Studies. Each sample was incubated at 25 °C under white light (1000 lx) produced by light-emitting diodes. Samples of the cells in the solutions were collected every two days until 8 d had passed. The cells in each sample were counted using the colony counting method using a blood cell calculator (DHC-N01; Air Brown).

The PAW treated for 120 min gave a good algicidal effect, so we prepared a solution to mimic the PAW solution. The mimic solutions were at pH 3 and contained NO_2_^−^ and H_2_O_2_. The mimic solutions were used in experiments to investigate the prevention of algal growth. A control and seven PAW mimic solutions were used as samples. The sample compositions are shown in Table 1. The pH was adjusted using nitric acid, and the NO_2_^−^ and H_2_O_2_ concentrations were 0.1 and 0.4 mmol L^−1^, respectively, to mimic the concentrations found in PAW after 120 min of plasma treatment. The other experimental conditions were the same as in the previous experiment that was performed to investigate the prevention of algal growth.

A basil seed was placed on top of an aliquot of each sample in a cup containing Styrofoam and medium to determine whether plants could be grown in PAW while preventing algal growth. A small amount of the test solution was added to the seed to moisten it, then the cup was filled with the solution. Adjusted Hoagland solution and the test solutions were used. The pH of the PAW treated for 120 min decreased to ∼3, so a pH adjustment agent (OAT Agrio) was used to adjust the pH to 6. The plants were grown for 10 d at 25 °C under white light (1000 lx) emitted by light-emitting diodes.

### Peroxynitrite quantification experiment

The pH in the vicinity of *Chlorella vulgaris* cells was measured using the pH-responsive microelectrodes. Each microelectrode was made as described previously.^50,51^ A copper wire (Nilaco) and a platinum wire (diameter 76 μm; Nilaco) were connected using silver paste (D-550; Fujikura Kasei). The connection was coated with UV-curing resin (3035B; ThreeBond) and then the resin was cured. The copper/platinum wire was then inserted into a capillary made from a Pasteur pipette. The capillary was filled with UV-curing resin (40416, Pajco), then the resin was cured. The platinum wire was exposed only at the tip to keep the tip as thin as possible. The tip was covered with UV-curing resin, then the resin was cured. The microelectrode was then finished by cutting the tip. The prepared electrodes were characterized using 0.1 M KCl aq. (Wako Pure Chemicals) containing 0.5 mM K_4_[Fe(CN)_6_] and 0.5 mM K_3_[Fe(CN)_6_] (Wako Pure Chemicals). A Ag/AgCl electrode (RE-1CP; ABS) was used as the reference electrode and a Pt mesh was used as the counter-electrode. CV measurements were performed. Iridium oxide, an inorganic metal oxide, was electrodeposited on the microelectrode to give a stable pH response even under strongly acidic conditions. The electrodeposition solution contained 2.0 mM K_2_IrCl_6_ aq. (Sigma-Aldrich), and 10 wt% NaOH aq. (Wako Pure Chemicals) was added to bring the solution to pH 13. The solution was then heated to 90 °C for 20 min and then cooled in ice before 3 M HNO_3_ aq. (Wako Pure Chemicals) was quickly added to the cold solution to bring the solution to pH 1. The solution was then stirred for 80 min. Finally, 10 wt% NaOH aq. was added to bring the solution to pH 13.^52^ UV-vis measurements were performed to confirm that IrOx·*n*H_2_O nanoparticles were dispersed in the electrodeposition solution. The scanning range was 750–350 nm, and the other conditions and the devices used were the same as for determining H_2_O_2_.

Electrodeposition was performed using the prepared electrodeposition solution and a scanning rate of 20 mV s^−1^. CV measurements were performed for 20 cycles. Before electropolymerization, the electrodes were cleaned by performing CV on 0.5 M H_2_SO_4_ at a scanning rate of 30 mV s^−1^. The prepared electrodes were characterized by performing CV measurements for 20 cycles using the prepared electrodeposition solution and a scanning rate of 20 mV s^−1^. Before electropolymerization, the electrodes were cleaned as described above. The responsiveness of the electrodes to the pH was evaluated by performing OCP measurements. A standard pH solution (HORIBA) and in-house buffer solutions were used as standards for preparing a calibration curve. The standard samples used are shown in Table 2. The in-house buffer solutions were at pH 5 and 3. The pH 5 buffer solution was prepared by mixing 1.54 mL L^−1^ acetic acid and 6.11 g L^−1^ sodium acetate, and the pH 3 buffer solution was prepared by mixing 0.42 mL L^−1^ phosphoric acid and 6.84 g L^−1^ sodium dihydrogen phosphate dihydrate. A pH 1 solution was prepared by adding nitric acid to ultrapure water.

**Table 2:** Solutions used as pH standards

Sample	Solution
pH = 7	Neutral phosphate standard solution
pH = 5	Acetic acid buffer
pH = 4	Phthalate standard solution
pH = 3	Acid phosphate buffer
pH = 2	Oxalate standard solution
pH = 1	Nitric acid aqueous solution

A standard pH solution was brought to 25 ± 5 °C in a thermostatically controlled bath, then the pH was measured using a pH meter before an OCP measurement was made. The prepared electrode was fixed using a manipulator and transferred to a Petri dish containing cultured *Chlorella vulgaris*. The dish was placed in a microscope (NDL-6LED; Carton) and C medium and a phosphate buffer solution were added. The electrode was sited so that it could be inserted into the Petri dish, and the position was adjusted using a manipulator while observing the position using the microscope so that the tip of the electrode was in contact with an algal aggregate. A Ag/AgCl electrode (RE-1CP; BAS) was used as the reference electrode and a Pt mesh was used as the counter-electrode. These were placed in the phosphate buffer solution in the Petri dish, as shown in Fig. S8.† The pH in the vicinity of the cells was then measured by performing OCP measurements.

Peroxynitrite was quantified using a fluorescent probe specific to peroxynitrite (DAX-J2TM PON Green99; AAT Bioquest). A calibration curve was prepared before using the fluorescent probe. A 1 mL aliquot of a peroxynitrite solution (unknown concentration; Dojin Chemical Research Institute) was diluted by a factor of 100 with 0.1 M NaOH aq. (Wako Pure Chemicals) and used as a stock solution. The stock solution was further diluted by factors of 2 and 5. The concentrations in the stock solution and diluted solutions were estimated from the intensities of the UV-vis spectrophotometry peaks at 300 nm. Fluorescence spectroscopy was performed after mixing 0.3 mL of a solution with 0.3 mL of the fluorescent probe. A peroxynitrite concentration–fluorescence intensity calibration curve was prepared. The UV-vis measurements were made using a scanning range of 450–200 nm, and the other conditions and equipment used were the same as for determining H_2_O_2_. The FP-8300 fluorescence spectrophotometer (Japan Spectroscopy) was used with a scanning speed of 500 nm min^−1^, a wavelength range of 500–650 nm, an integration time of 2, an excitation wavelength of 490 nm, an excitation bandwidth of 5 nm, a fluorescence bandwidth of 10 nm, and a Xe light source. A peroxynitrite concentration–fluorescence intensity calibration curve was prepared, then the pH range in which peroxynitrite could be produced was identified using a fluorescent probe. A 0.1 mL aliquot of HCl aq. (Wako Pure Chemicals) at pH 1, 2, 3, 5, or 7 was mixed with 0.1 mL of 0.6 M H_2_O_2_ aq. (Wako Pure Chemicals). A 0.1 mL aliquot of 0.6 M NaNO_2_ aq. (Wako Pure Chemicals) was then added, then 3 M NaOH aq. (Wako Pure Chemicals) was quickly added.^52^ A 0.4 mL aliquot of the fluorescent probe was then added, and the solution was subjected to fluorescence spectroscopy.^53^ The fluorescence bandwidth was 10 nm, and the other conditions and equipment used were the same as for preparing the calibration curve.

## Conclusions

A batch-type in-liquid plasma system was used to produce PAW that prevented algae growing. NO_2_^−^, NO_3_^−^, and H_2_O_2_ in the PAW were found to be critical to the algicidal effect, and the peroxynitrite produced by the reactions between these active species played an important role in the algicidal effect. The pH near algal cells was measured using an iridium oxide microelectrode, and the solution in the vicinity of each cell was found to be very acidic (pH 2.23). Peroxynitrite could be generated in this area because it can be produced at pH 1–3. The PAW can be used as a liquid fertilizer to grow plants while preventing algal growth, and it could be used to prevent contamination of the solutions and cultivation tanks used in plant growing factories. The results indicated that it will be possible to develop an environmentally friendly disinfectant using common materials. The PAW could contribute to solving food shortages and sustainable development.

## Author contributions

KM, MS, SS and KH: data curation, formal analysis, investigation. KM and VRG: writing-original draft. NI, NS, KK, TK and MY: investigation, methodology. AF and KT: conceptualization, supervision. CT: conceptualization, funding acquisition, project administration, supervision.

## Conflicts of interest

There are no conflicts of interest to declare.

## Supplementary Material

RA-012-D1RA07774K-s001
