# Supplementary material for: Interactions between pH, reactive species, and cells in plasma-activated water can remove algae
Source: RSC Adv. 2022 Mar 8;12(13):7626–34. doi: 10.1039/d1ra07774k (PMC8982187; doi:10.1039/d1ra07774k)
Supplement: RA-012-D1RA07774K-s001 [file RA-012-D1RA07774K-s001.pdf]

Electronic Supplementary Material (ESI) for RSC Advances.
This journal is © The Royal Society of Chemistry 2022

Supporting Information

Using the two-line intensity comparison method, the electron temperature was calculated from the intensity ratio of the two lines at H_α (656 nm) and H_β (486 nm)[1)]. Since the spectral intensity of a line is expressed in terms of the upper level density N, transition probability A, and energy per photon hv, the intensity of the line spectrum at each transition is as follows.

$$I_{ij} = h\nu_{ij}A_{ij}N_i \qquad (1)$$

$$I_{kl} = h\nu_{kl}A_{kl}N_k \qquad (2)$$

When discharged at high pressure, such as atmospheric pressure, the collisions between electrons, ions, and neutral particles are so intense that the particles exchange enough kinetic energy to reach thermal equilibrium. At this time, the electron temperature T_e, ion temperature T_i, and neutral particle temperature T_n are equal, as shown in equation (3).

$$T = T_e = T_i = T_n \qquad (3)$$

Since the distribution of the density of each level is expressed by the Boltzmann distribution, the density ratio is as shown in equation (4).

$$\frac{N_i}{N_k} = \frac{g_i}{g_k} exp\left\{ - \frac{E_i - E_k}{kT_e} \right\} \qquad (4)$$

By transforming equation (5), equation (6) can be obtained. The values used in the calculations are shown in the Table S1.

I: Line spectral intensity

g: statistical weight

ν: Frequency (s^{-1})

A: Einstein coefficient (s^{-1})

T_e: electron temperature (K)

K: Boltzmann constant (J/K)

E: Potential energy (J)

References

1) J. Yamada, *Plasma and Fusion Research*, 1993, **69**, 784-792

Fig.S1 A basil growth test to investigate the possibility of using PAW for plant growth
Fig.S2 CV curves for electrodeposited iridium oxide
Fig.S3 PH response gradient of iridium oxide electrodeposited electrodes (n=7)
Fig.S4 Reversibility evaluation in pH responsiveness of electrodeposited iridium oxide electrodes
Fig.S5 Fluorescence measurement results when peroxynitrite was generated under each pH condition
Fig.S6 Identifying the pH range in which peroxynitrite can be generated
Fig.S7 The schematic diagram of batch type in-liquid plasma system
Fig.S8 Experimental system for measuring pH in the near of cells

Table S1 Value used to calculate electron temperature
Table S2 Power condition, Hα, Hβ and electron temperature corresponding to entry
Table S3 Mixing ratio of various stock solutions for Hoagland solution adjustment

Fig.S1

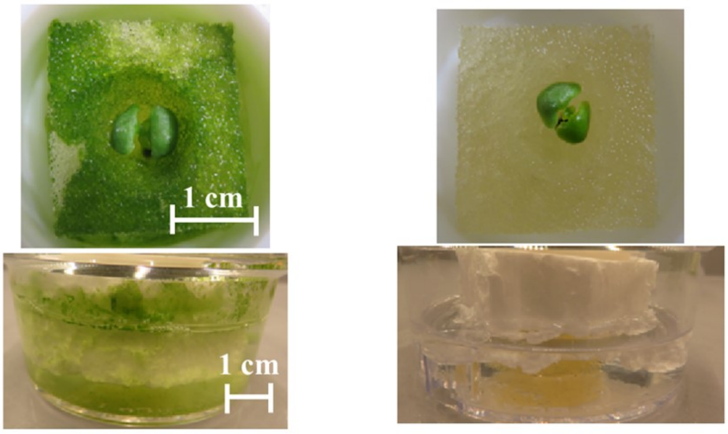

(a) The Hoagland solution **(b) Plasma activated water**

Fig.S2

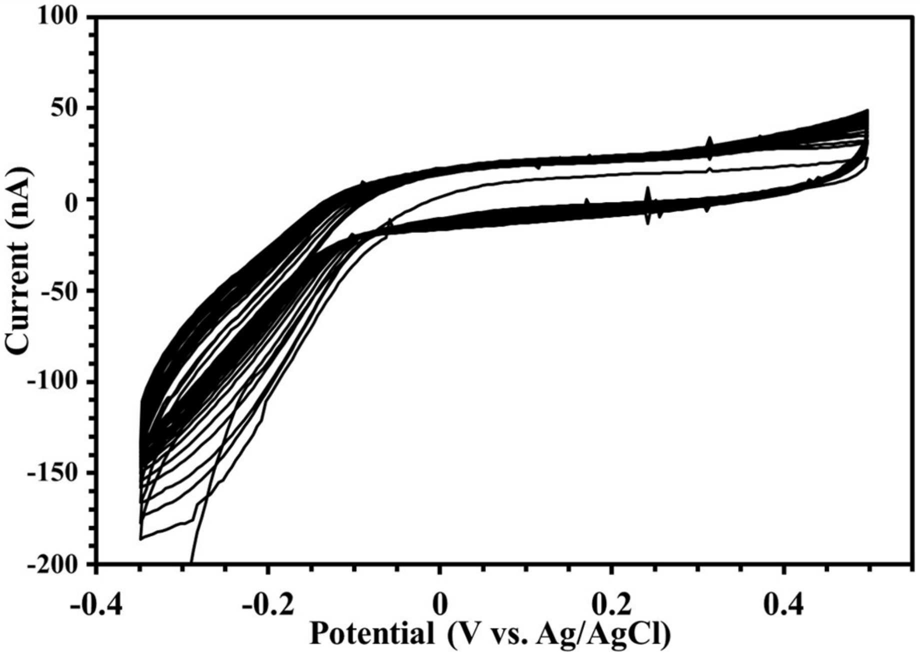

100
50
0
-50
-100
-150
-200
Current (nA)
-0.4
-0.2
0
0.2
0.4
Potential (V vs. Ag/AgCl)

Fig.S3

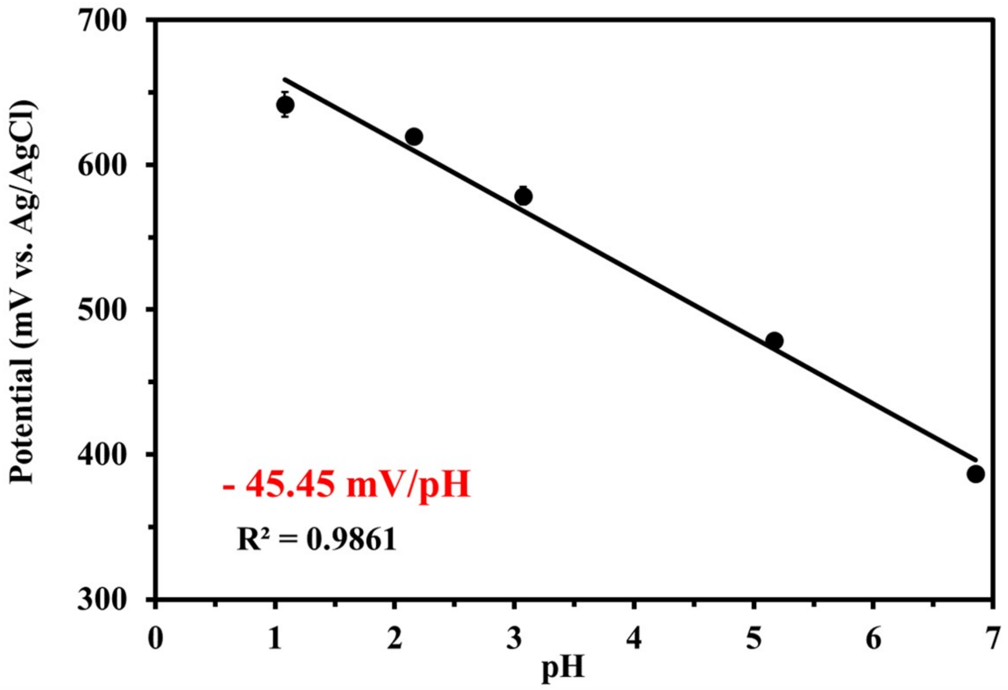

Potential (mV vs. Ag/AgCl)
700
600
500
400
300
0
1
2
3
4
5
6
7
pH
- 45.45 mV/pH
R² = 0.9861

Fig.S4

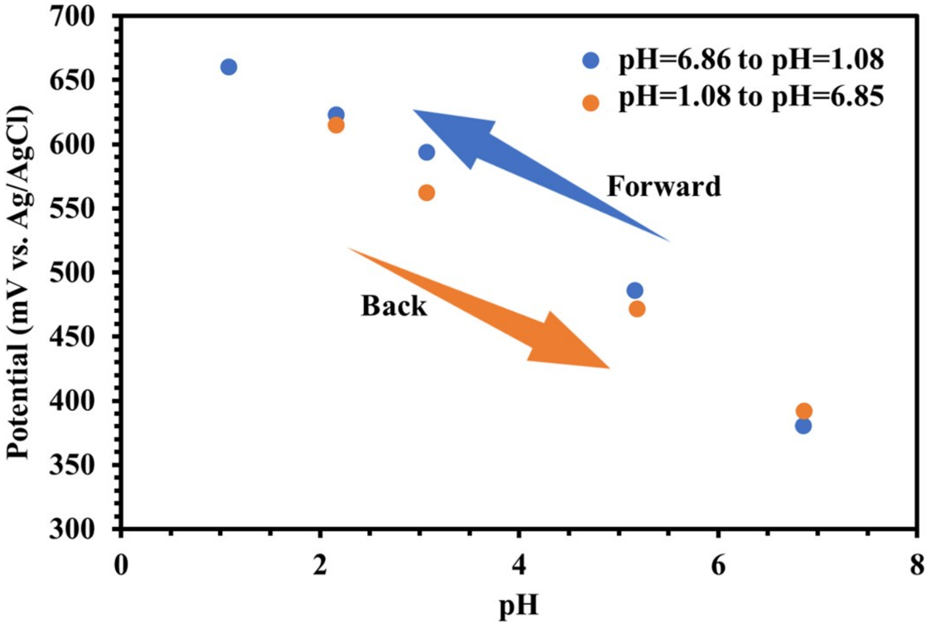

Potential (mV vs. Ag/AgCl)
700
650
600
550
500
450
400
350
300
0
2
4
6
8
pH
pH=6.86 to pH=1.08
pH=1.08 to pH=6.85
Forward
Back

Fig.S5

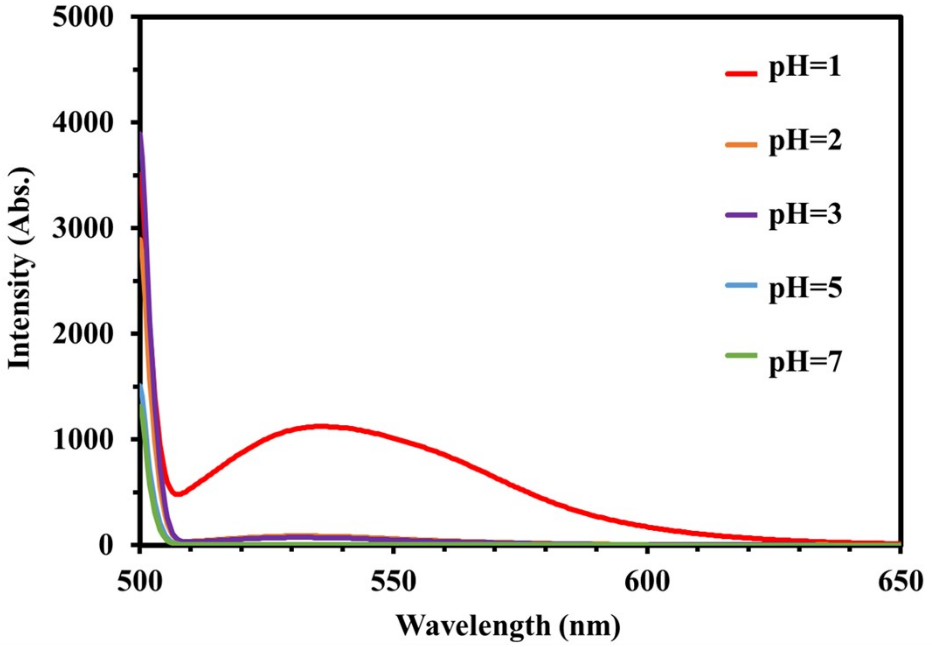

5000
4000
3000
2000
1000
0
Intensity (Abs.)
500
550
600
650
Wavelength (nm)
pH=1
pH=2
pH=3
pH=5
pH=7

Fig.S6

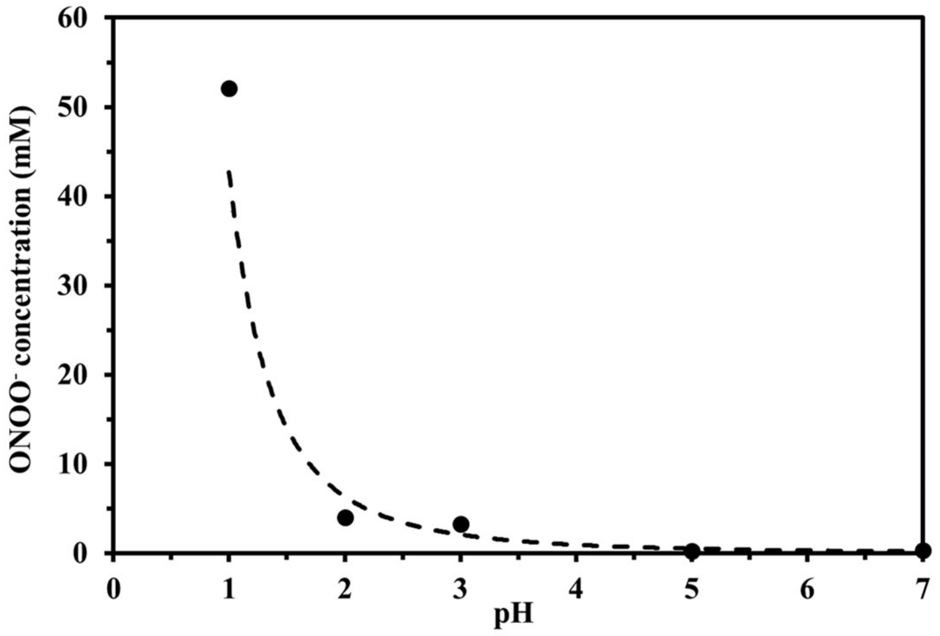

Fig.S7

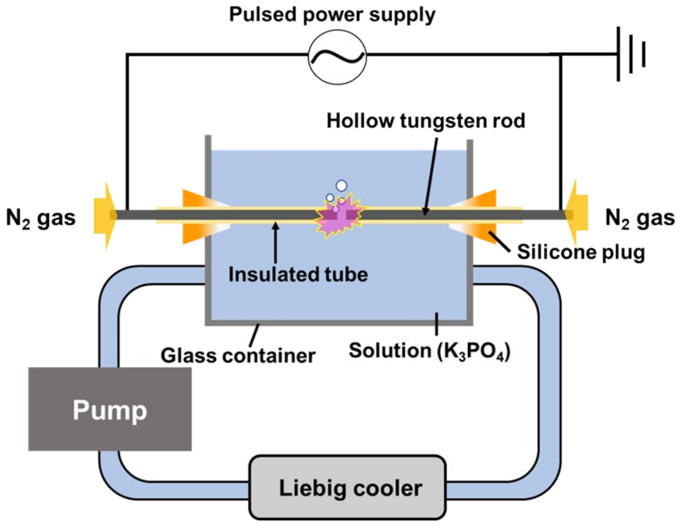
Pulsed power supply
Hollow tungsten rod
N2 gas
N2 gas
Silicone plug
Insulated tube
Glass container
Solution (K3PO4)
Pump
Liebig cooler

Fig.S8

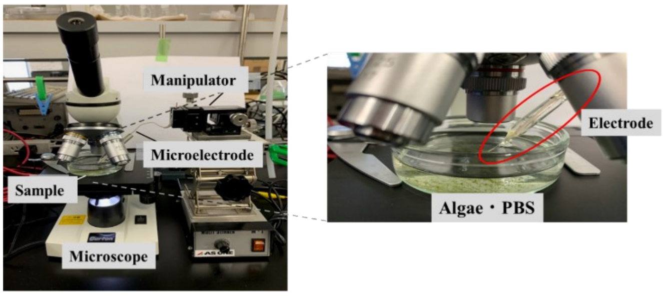
Manipulator
Microelectrode
Sample
Microscope
Electrode
Algae・PBS

Table S1

Entry	Content		
	pH=3	NO_2^-	H_2O_2
1	○	○	○
2	○	×	○
3	○	○	×
4	×	○	○
5	○	×	×
6	×	×	○
7	×	○	×

Table S2

Entry	Frequency (kHz)	Pulse Width (μsec)	H_α Intensity	H_β Intensity	Electron temperature (eV)
1	20	0.18	37963	5686	0.739
2	20	0.38	274789	42009	0.726
3	50	0.18	4931	722	0.754
4	50	0.38	232097	25667	0.940
5	100	0.18	174667	15479	1.09
6	100	0.38	357748	46969	0.827
7	120	0.18	148314	12846	1.10
8	120	0.38	357743	69730	0.565
9	150	0.18	224096	16773	1.20
10	200	0.18	325009	27080	1.13

Table S3

Sample	Content (g/L)	Mixture quantity (mL)
H1	KNO_3 : 101 g/L (= 1 M)	0.5
H2	Ca (NO_3)・4H_2O : 236 g/L (= 1 M)	0.2
H3	$MgSO_4$・7H_2O : 246 g/L (= 1 M)	0.1
H4	$NH_4H_2PO_4$: 115 g/L (= 1 M)<br>H_3BO_3 : 1.85 g/L (= 0.03 M)<br>$MnCl_2$・4H_2O : 0.99 g/L (= 0.005 M)<br>(NH_4)6Mo_7O_{24}・4H_2O : 12.36 g/L (= 0.01 M)<br>$ZnSO_4$・7H_2O : 1.15 g/L (= 0.004 M)	0.01
Fe	Fe (Ⅲ)-EDTA : 82.4 g/L (= 0.2 M)	0.01
Cu	$CuSO_4$・5H_2O : 0.50 g/L (= 0.002 M)	0.01
